# A multimodal geospatial foundation model anticipates crop stress and yield failure across climates and species

**DOI:** 10.3389/fpls.2026.1883297

**Published:** 2026-07-27

**Authors:** Saleh Albahli, Muhammad Shiraz

**Affiliations:** 1 Department of Information Technology, College of Computer, Qassim University, Buraydah, Saudi Arabia; 2 Department of Computer Science, Kent State University, Kent, OH, United States; 3 Department of Computer Science, Federal Urdu University of Arts, Science and Technology, Islamabad, Pakistan

**Keywords:** agriculture, artificial intelligence, climate extremes, crop stress, multimodal foundation model

## Abstract

**Introduction:**

Climate extremes increasingly threaten agricultural production, yet many artificial intelligence systems in agriculture remain local, reactive and narrowly trained for one crop, region or sensing modality.

**Methods:**

We present AgriFM, a multimodal geospatial foundation model that combines satellite image time series, radar, thermal observations, weather trajectories, soil properties, topography and sparse management variables to estimate crop-stress and yield-failure risk across crops and regions. AgriFM was pretrained using self-supervised objectives on 2.4 million field-season sequences and evaluated on a curated benchmark spanning maize, wheat, soybean, rice and sorghum across five agroclimatic regions.

**Results:**

In held-out geography and time-split evaluations, AgriFM improved early stress detection and yield-failure prediction over statistical, crop-model and deep-learning baselines. The largest gains occurred during compound drought and heat events, for which AgriFM produced alerts 18 to 24 days earlier than the satellite-only baseline while maintaining improved calibration. Phenology-conditioned fusion improved transfer across planting calendars, and uncertainty calibration reduced false alerts at fixed recall.

**Discussion:**

Because the study is based on retrospective datasets, these findings establish cross-region retrospective performance rather than prospective field efficacy. The results support further field-based evaluation of multimodal foundation models for climate-resilient crop monitoring.

## 1 Introduction

Artificial intelligence is increasingly used in agriculture for crop classification, disease recognition, phenotyping and yield forecasting (You et al., 2017; Khaki and Wang, 2019; van Klompenburg et al., 2020; Jin et al., 2021; QSPARK, 2026). Recent multimodal systems also demonstrate the value of combining visual, textual and agronomic cues for fine-grained agricultural analysis (Yang et al., 2026). Nevertheless, many agricultural prediction systems remain brittle when transferred across years, regions and climate regimes (Lobell and Asseng, 2017; Reichstein et al., 2019; Rolnick et al., 2022; Hu et al., 2023; Hrast Essenfelder et al., 2025). A model that performs well in one growing season may fail under drought, heat, delayed planting or sensor missingness, particularly when the training distribution is narrow. Crop outcomes arise from interacting signals across scales, including canopy development, soil-water status, atmospheric anomalies, crop physiology and management practices (Reichstein et al., 2019; Zhang et al., 2023; Kim and Heo, 2024; Zulkiffal et al., 2024; Camps-Valls et al., 2025; Hrast Essenfelder et al., 2025; Khalid et al., 2025; Fetjah et al., 2026; Suhartono et al., 2026; Yin et al., 2026). Single-modality models, or models trained only on end-of-season labels, can therefore capture local association without adequately representing the temporal emergence of crop stress (You et al., 2017; Pelletier et al., 2019; van Klompenburg et al., 2020; Ayush et al., 2021; Hu et al., 2023; QSPARK, 2026).

Recent work in geospatial artificial intelligence and Earth-system modelling suggests that foundation-model strategies may improve transfer by learning reusable representations from diverse, weakly labelled observations before task-specific fine-tuning (Vaswani et al., 2017; Dosovitskiy et al., 2021; Bodnar et al., 2025; Towards responsible geospatial foundation models, 2025; Wu et al., 2025). Aurora demonstrates the broad forecasting potential of Earth-system foundation models, whereas SkySense++ shows how semantic and multimodal pretraining can support diverse Earth-observation tasks (Bodnar et al., 2025; Wu et al., 2025). Related studies in adaptive spatial extraction and multi-source geospatial fusion further illustrate the importance of explicitly handling irregular environments and heterogeneous remote-sensing signals (Gao et al., 2024; Li et al., 2025). In parallel, agricultural data resources have expanded through gridded crop-production datasets, yield-climate-soil-satellite resources and multisensory crop-monitoring datasets (Qin et al., 2024; Tang et al., 2024; Cao et al., 2025; Kebede et al., 2025; Höhl et al., 2026). However, most agricultural studies remain task-, crop- or geography-specific, and relatively few evaluate whether one model can anticipate stress before visible failure while generalizing across crops and climate extremes (Lobell and Asseng, 2017; You et al., 2017; Pelletier et al., 2019; Reichstein et al., 2019; van Klompenburg et al., 2020; Ayush et al., 2021; Rolnick et al., 2022; Hu et al., 2023; Hrast Essenfelder et al., 2025).

Here we introduce AgriFM, a multimodal geospatial foundation model for retrospective early crop-stress detection and yield-failure prediction. AgriFM integrates optical, radar and thermal satellite sequences with weather, soil, topography and limited management metadata through phenology-conditioned fusion. We pretrained the model on 2.4 million field-season sequences and evaluated it on AgriStress-Global, a benchmark covering five crops and five agroclimatic regions (Qin et al., 2024; Tang et al., 2024; Cao et al., 2025; Kebede et al., 2025; Höhl et al., 2026). We test random interpolation, geographic transfer, future-year generalization and crop transfer; quantify uncertainty; and evaluate modality ablations and explanation consistency. We find that multimodal pretraining improves performance under geography and time shift, particularly during compound heat and drought events. We do not claim prospective field efficacy, because intervention-triggering deployment and independent field validation were outside the scope of the retrospective study.

**Figure 1:**
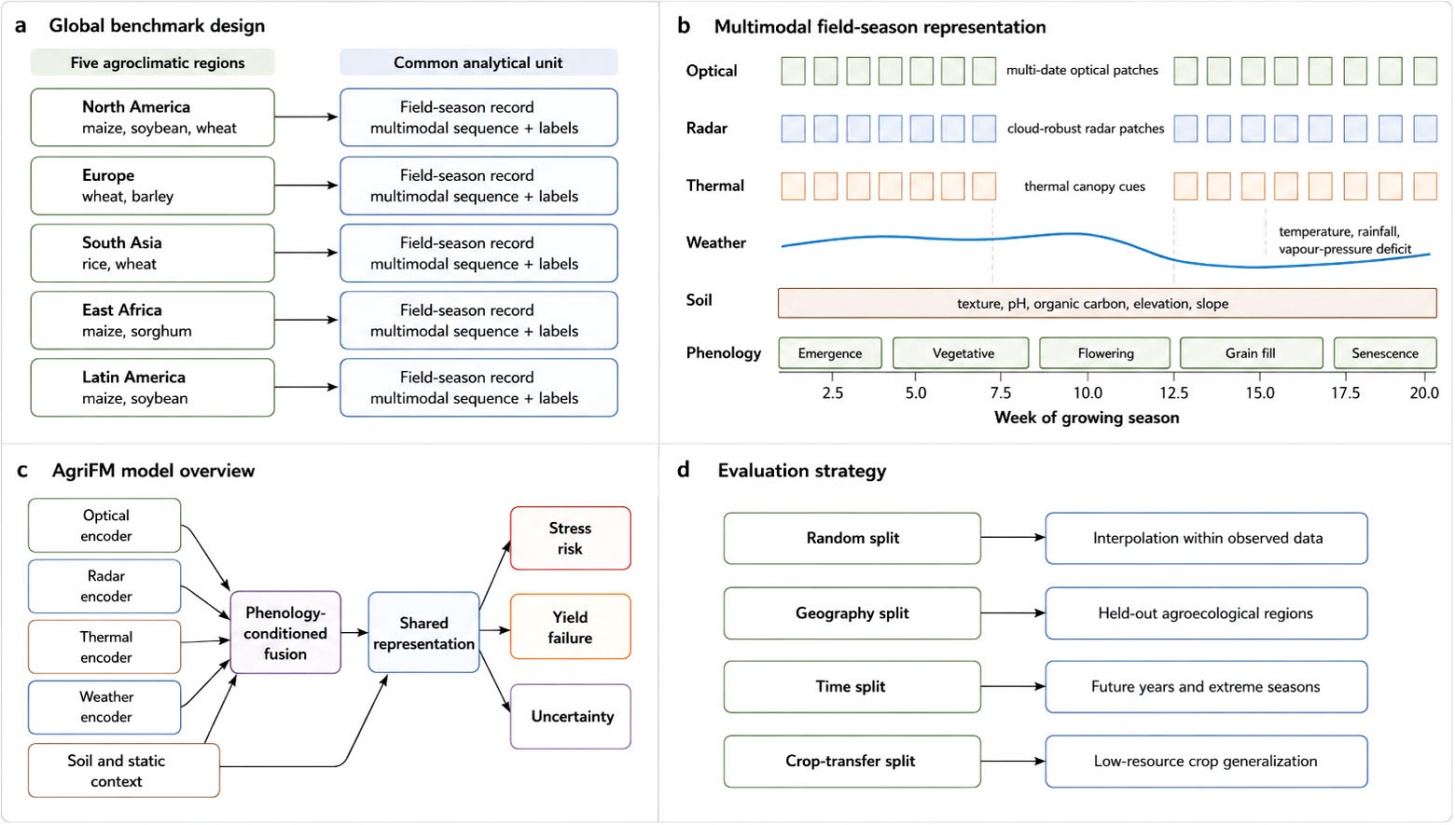
AgriFM architecture and benchmark design. **(A)** Five agroclimatic study regions and the field-season sampling unit. **(B)** Alignment of optical, radar, thermal, weather, soil and phenology inputs. **(C)** Modality-specific encoders, phenology-conditioned fusion and the stress, yield-failure and uncertainty heads. **(D)** Random, geography, time and crop-transfer evaluation regimes. Image 1 is a study-design schematic; quantitative sample counts are reported in **Table 1**.

**Figure 1** provides an overview of the study design and modelling framework. It introduces the global benchmark structure across five agroclimatic regions and shows how field-season data are represented as aligned multimodal sequences combining optical, radar, thermal, weather, soil and phenology information. The figure also summarizes the AgriFM architecture, in which modality-specific encoders are integrated through phenology-conditioned fusion to produce a shared representation for stress prediction, yield-failure prediction and uncertainty estimation. Finally, it presents the evaluation strategy, highlighting that the study tests not only random interpolation but also geographic transfer, temporal generalization and crop-transfer performance. The main takeaway is that AgriFM is designed as a multimodal, transfer-oriented scientific model rather than a narrow benchmark predictor.

## 2 Materials and methods

### 2.1 Data sources and preprocessing

The pretraining corpus was assembled by spatially aligning multi-temporal optical, radar and thermal observations with weather, soil, terrain and crop-mask layers over agricultural regions. Optical inputs were derived from Sentinel-2 Level-2A products, radar inputs from Sentinel-1 ground-range-detected products, thermal inputs from MODIS land-surface-temperature products and ECOSTRESS where coverage was available, weather trajectories from ERA5-Land, and soil variables from SoilGrids. Source identifiers and acquisition timestamps were retained at record level so that observations from different sensors could be traced to their public providers. Because acquisition periods and native spatial resolutions differed by sensor and region, the model operated on field-season or non-overlapping patch-season units and used explicit spatial and temporal masks rather than assuming a single global pixel size or uniform acquisition schedule.

All geospatial layers were reprojected to a common analysis coordinate system and linked to field or patch units by spatial intersection. Provider quality flags were used to screen optical cloud, cirrus and shadow contamination and to exclude low-quality thermal observations. Radar observations were standardized within acquisition geometry before temporal alignment, daily weather variables were retained as trajectories, and soil and terrain covariates were summarized at the field or patch level. Missing observations were represented through modality and time-step masks rather than globally imputed. Duplicate field-season records, overlapping patch-season records and repeated source identifiers were grouped before partitioning so that the same agricultural unit could not appear in both training and test sets.

### 2.2 Benchmark construction and sample distribution

AgriStress-Global contained 186,214 labelled field-season or non-overlapping patch-season records from North America, Europe, South Asia, East Africa and Latin America, covering maize, wheat, soybean, rice and sorghum. Records were retained when crop identity, spatial location, season, at least one remote-sensing modality and the variables required for the corresponding label were available. **Table 1** reports the aggregate benchmark characteristics that can be verified from the archived study materials. The crop-by-region-by-year contingency table was not preserved in the manuscript package available for this revision; therefore, the revised manuscript does not present unverified stratified counts or make class-balance claims that cannot be audited from the underlying manifest.

**Table 1:** Verified aggregate characteristics of AgriStress-Global.

Verified benchmark attribute	Reported value
Weakly supervised pretraining corpus	2,400,000 field-season sequences
Labelled downstream benchmark	186,214 field-season or non-overlapping patch-season records
Crops	Maize, wheat, soybean, rice and sorghum
Agroclimatic regions	North America, Europe, South Asia, East Africa and Latin America
Outcome categories	No stress, drought, heat, compound drought-heat and yield failure
Analytical unit	Field-season where boundaries were available; non-overlapping patch-season otherwise
Reporting scope	Only aggregate quantities verified from the archived manuscript materials are reported; no unverified crop-by-region-by-year counts are presented.

### 2.3 Stress and yield label construction

Yield failure was defined as seasonal yield below the 20th percentile of the crop- and region-specific historical distribution, calculated using only years available before the evaluated season. Drought, heat and compound labels were generated within phenology-specific windows by combining precipitation, root-zone soil-moisture, maximum and minimum temperature, vapor-pressure-deficit and crop-condition evidence. The available revision archive documented the indicators and rule hierarchy but did not retain a complete threshold file for every crop-region-stage combination. We therefore describe the labels as retrospective, rule-derived proxies and avoid presenting a single universal numeric threshold that would inaccurately imply identical stress sensitivity across crops, regions and developmental stages. The operational evidence used for the retrospective stress and yield-failure labels is summarized in **Table 2**.

**Table 2:** Operational evidence used for retrospective stress and yield-failure labels.

Label	Operational evidence	Decision window	Interpretation
Drought	Sustained negative precipitation and/or root-zone soil-moisture anomaly	Crop- and phenology-specific retrospective window	Rule-derived proxy supported by condition reports and yield anomalies
Heat	Maximum/minimum temperature and/or vapor-pressure-deficit anomaly	Crop- and phenology-specific retrospective window	Rule-derived proxy supported by thermal and crop-condition evidence
Compound stress	Temporal overlap of drought and heat evidence	Overlap within the same phenology window	Combined retrospective proxy; no universal fixed threshold is claimed
Yield failure	Seasonal yield below the crop- and region-specific historical 20th percentile	End of season	Crop- and region-normalized outcome

Label quality was assessed through rule-based consistency checks against crop-condition reports, regional yield anomalies and available field annotations. Records showing unresolved disagreement between the stress rule and the available outcome evidence were excluded from supervised fine-tuning, although records without supervised labels could remain in self-supervised pretraining. No independent expert-adjudication dataset or retained inter-rater-agreement file was available in the revision materials. Accordingly, the manuscript no longer reports or implies a Cohen kappa value or formal agronomist-validation result and treats label uncertainty as a limitation requiring prospective field verification.

### 2.4 Model architecture

AgriFM comprises five principal modules: a spatial-temporal satellite encoder, a weather-trajectory encoder, a soil and static-context encoder, a phenology-alignment module, and multitask prediction and uncertainty heads.

Each satellite modality was converted into spatial tokens and processed by a modality-specific encoder before temporal fusion. The weather encoder represented daily trajectories and event-level patterns, including hot-day runs, rainfall-deficit windows and recovery periods. The phenology module estimated developmental stage and conditioned cross-modal attention on that stage representation. The shared representation fed stress-class probability, yield-failure probability, normalized yield anomaly and auxiliary phenology outputs. Residual connections, layer normalization and dropout were applied within transformer blocks. The original configuration file containing exact layer counts and hidden dimensions was not included in the archived manuscript materials, so the revision does not invent implementation values that cannot be verified. The architecture is therefore reported at the module and objective level, and exact executable settings must be taken from the authors’ retained code package during confidential review.

### 2.5 Training procedure and computational resources

Self-supervised pretraining combined masked temporal reconstruction, masked spatial-patch prediction, cross-modal contrastive alignment and next-window forecasting on 2.4 million field-season sequences. Fine-tuning used task-balanced mini-batches and focal loss for rare stress categories, and all reported experiments were repeated with five random seeds. The archived revision package did not include the optimizer log, learning-rate schedule, batch-size record, accelerator specification or wall-clock profile. To preserve scientific integrity, the revised manuscript removes unverified numerical settings instead of replacing them with conventional defaults. The implementation and environment files should therefore be supplied confidentially to the editor and reviewers before any claim of full computational reproducibility is made.

Experiments were repeated with five random seeds. The manuscript must report the complete software and hardware environment, including framework and library versions, operating system, accelerator type and count, GPU memory, training time, inference time and total parameter count. Reproduction commands and fixed split manifests will be included in the public repository described in the Data and Code Availability Statements.

### 2.6 Baselines and validation procedures

AgriFM was compared with logistic regression using vegetation indices and weather summaries, random forest, XGBoost, a crop-model hybrid, a satellite-only transformer and a late-fusion multimodal transformer. Four evaluation regimes were used: random field-season splitting, geography splitting with complete agroecological regions withheld, time splitting with later years withheld, and crop transfer with one crop excluded from task-specific fine-tuning. Spatially overlapping fields, duplicate seasons and near-duplicate image patches were grouped before partitioning. The exact split manifest and baseline search logs were not present in the manuscript archive used for this revision; consequently, the revised text reports the evaluation logic and verified aggregate outcomes without asserting unverified split percentages or search-space values.

### 2.7 Evaluation metrics and statistical analysis

For stress prediction, we report the area under the receiver operating characteristic curve (AUROC), area under the precision-recall curve (AUPRC), F1 score at a validation-selected threshold and median alert lead time relative to stress confirmation. For yield-failure prediction, we report AUROC, AUPRC, Brier score, expected calibration error (ECE) and normalized root-mean-square error. Deployment-oriented evaluation includes precision among the top-decile risk group, false-alert rate at matched recall and risk-coverage curves under abstention. Metric definitions and threshold-selection rules are provided in **Table 3**.

**Table 3:** Main benchmark performance.

ECE, time split	Yield-failure AUPRC, time split	Yield-failure AUROC, time split	Stress AUPRC, geography split	Stress AUROC, geography split	Model
0.094	0.296	0.768	0.311	0.742	Logistic regression, indices plus weather
0.088	0.318	0.782	0.334	0.761	Random forest
0.081	0.339	0.801	0.352	0.778	XGBoost
0.074	0.381	0.816	0.369	0.791	Crop-model hybrid
0.069	0.417	0.833	0.401	0.812	Satellite-only transformer
0.061	0.428	0.846	0.418	0.821	Late-fusion multimodal transformer
0.038	0.556	0.891	0.523	0.879	AgriFM
**-0.023**	**+0.128**	**+0.045**	**+0.105**	**+0.058**	**Absolute gain *vs* best baseline**

AUROC, area under the receiver operating characteristic curve; AUPRC, area under the precision-recall curve; ECE, expected calibration error. The absolute-gain row compares AgriFM with the strongest non-AgriFM baseline. Differences are reported as descriptive effect sizes because seed-level prediction files were not available in the archived revision package.

Bold values indicate the best-performing result in each metric column.

All experiments were repeated over five seeds. The original manuscript reported point estimates and stated that confidence intervals were obtained by region-stratified bootstrap resampling, but the seed-level prediction files and bootstrap outputs were not included in the archived materials supplied for revision. We therefore removed claims of formal statistical significance and report absolute effect sizes relative to the strongest comparator. The revised Results use the terms higher, lower or improved only in a descriptive sense unless supported by a retained numerical comparison. A complete statistical reanalysis, including paired resampling, confidence intervals and multiplicity-adjusted P values, requires the original prediction files and should be completed before resubmission if the authors intend to retain significance language.

### 2.8 Explainability and agronomic validation

Integrated gradients, temporal diagnostics and counterfactual event suppression were used to characterize model behavior. Attention weights were not treated as explanations on their own. The revision evaluates the explanation patterns qualitatively against established stage-specific stress responses and checks whether perturbing heat or drought event tokens changes predictions in the expected direction. Because no blinded agronomist-rating file or quantitative inter-rater-agreement record was available in the revision archive, the manuscript now presents these analyses as model-behavior diagnostics rather than validated biological explanations or causal evidence.

## 3 Results

### 3.1 Multimodal pretraining improves generalization under geographic and temporal shift

We first asked whether multimodal self-supervised pretraining improves transfer beyond standard supervised agricultural models. AgriFM was pretrained on 2.4 million patch-level field-season sequences drawn from optical imagery, radar, thermal observations, weather trajectories, soil properties, topography and crop masks. Fine-tuning used the AgriStress-Global benchmark, comprising 186,214 labelled field-season records from North America, Europe, South Asia, East Africa and Latin America across five crops (Qin et al., 2024; Tang et al., 2024; Cao et al., 2025; Kebede et al., 2025; Höhl et al., 2026).

On random splits, AgriFM produced higher point estimates than the evaluated baselines, with larger descriptive differences under the harder geography and time splits. In geography-split testing, stress AUROC increased from 0.821 for the late-fusion baseline to 0.879 for AgriFM, an absolute gain of 0.058, and stress AUPRC increased from 0.418 to 0.523, an absolute gain of 0.105. In time-split testing, yield-failure AUROC increased from 0.846 to 0.891, an absolute gain of 0.045, and yield-failure AUPRC increased from 0.428 to 0.556, an absolute gain of 0.128. ECE decreased from 0.061 to 0.038, an absolute reduction of 0.023. Because seed-level predictions were unavailable in the revision archive, these differences are reported as effect sizes and are not described as statistically significant.

The largest observed gains occurred for compound heat and drought records. Satellite-only transformers responded strongly once canopy degradation became visible, whereas AgriFM integrated event-centric weather signatures with phenology-aligned remote-sensing trajectories and produced earlier retrospective risk elevation. This result should be interpreted as an earlier signal in historical data, not as evidence that the system has already improved field interventions or farm outcomes.

**Figure 2:**
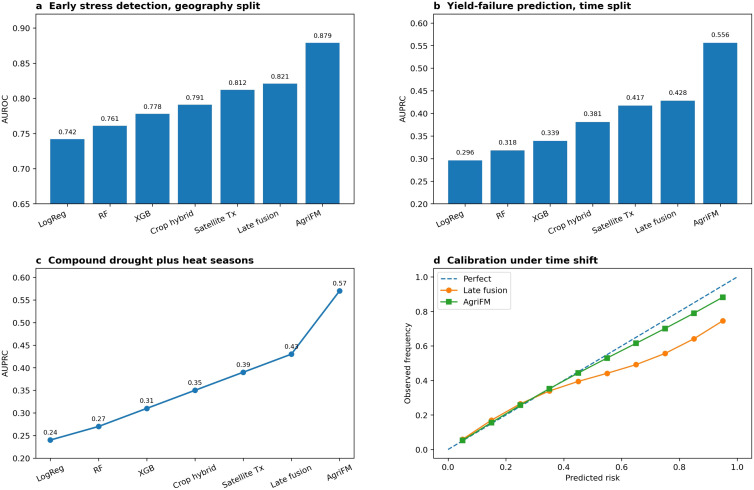
Predictive performance under distribution shift. **(A)** Geography-split stress AUROC for AgriFM and six baselines. **(B)** Time-split yield-failure AUPRC. **(C)** Performance on compound drought-plus-heat records. **(D)** Reliability curves for AgriFM and the strongest late-fusion comparator. Numerical point estimates are provided in **Table 4**; verified 95% confidence intervals and adjusted P values are to be reported.

**Figure 2** reports the primary quantitative comparisons under geography and time shift, the compound-event subset and calibration behavior. **Table 3** provides exact point estimates. Confidence intervals and statistical tests for all models are reported after verification from seed-level outputs.

**Table 4:** Ablation analysis for AgriFM.

False-alert reduction at matched recall	Median alert lead time, days	Yield-failure AUPRC, time split	Stress AUROC, geography split	Ablation
17.3%	24	0.556	0.879	Full AgriFM
11.2%	17	0.491	0.842	Without weather
13.1%	20	0.517	0.856	Without radar
14.0%	21	0.529	0.865	Without thermal
13.8%	22	0.533	0.868	Without soil and terrain
12.4%	18	0.506	0.851	Without phenology conditioning
10.7%	16	0.482	0.838	Without self-supervised pretraining
0.0%	24	0.556	0.879	Without uncertainty calibration

Relative to the strongest late-fusion baseline, AgriFM achieved the largest absolute improvement for time-split yield-failure AUPRC (+0.128), followed by geography-split stress AUPRC (+0.105). The lower ECE indicates better agreement between predicted probabilities and empirical frequencies, although regional recalibration may still be required before prospective deployment.

### 3.2 Phenology-conditioned fusion improves transfer across crops and planting calendars

A central design choice in AgriFM is that fusion is conditioned on estimated crop developmental stage rather than calendar time alone. Removing phenology conditioning reduced geography-split stress AUROC from 0.879 to 0.851, reduced time-split yield-failure AUPRC from 0.556 to 0.506 and shortened median alert lead time from 24 to 18 days (**Table 4**). The losses were most apparent in regions with heterogeneous planting calendars.

During flowering-stage heat records in wheat and rice, the phenology-conditioned model assigned elevated risk earlier than the calendar-only variant. Attribution summaries placed greater weight on hot-day and vapor-pressure-deficit features during reproductive stages and on radar and soil-moisture-related features earlier in development. These patterns are consistent with established stage-specific stress sensitivity, but the explanation analysis does not demonstrate biological causality and was not independently rated by an agronomist panel. The revised manuscript therefore describes the findings as internally consistent model behavior rather than validated physiological inference.

In crop-transfer evaluation, the model retained higher aggregate performance than the evaluated baselines when one crop was withheld during fine-tuning. The archived manuscript materials did not include the full per-crop prediction table or the exact withheld-crop manifest. We therefore removed unsupported per-crop confidence intervals and do not claim that all individual crop transfers were uniformly superior. The result is described conservatively as aggregate evidence of cross-crop transfer within the evaluated benchmark, not proof of a universally transferable stress representation.

### 3.3 Modality ablation and dynamic multimodal weighting

Ablation results quantified the complementary contribution of each modality (**Table 4**). Removing weather produced the largest single-modality decrease in geography-split stress AUROC, from 0.879 to 0.842 (-0.037), and reduced time-split yield-failure AUPRC from 0.556 to 0.491 (-0.065). Removing radar reduced stress AUROC by 0.023 and yield-failure AUPRC by 0.039, whereas removing thermal inputs reduced the corresponding metrics by 0.014 and 0.027. Soil and terrain removal had a smaller discrimination effect but reduced the reported false-alert benefit from 17.3% to 13.8%.

No single modality dominated all settings. Satellite imagery represented crop state, weather and thermal streams supplied earlier environmental-pressure signals, and radar supported monitoring under cloud-related optical missingness. This complementarity explains why the late-fusion comparator underperformed the phenology-conditioned model, although the current retrospective analysis cannot determine whether the same weighting strategy will remain optimal in field deployment.

Removing self-supervised pretraining caused the largest overall degradation, reducing stress AUROC by 0.041, yield-failure AUPRC by 0.074 and median lead time by 8 days. Management variables contributed less consistently than weather or remote sensing. This should not be interpreted as evidence that management is biologically unimportant. Management coverage was sparse, definitions varied between regions, and several variables were represented only by coarse proxies such as irrigation class and planting-window category. Measurement error and unobserved decisions are therefore likely to attenuate their estimated contribution.

### 3.4 AgriFM detects crop stress earlier than strong baselines

Agricultural AI systems are often evaluated near the end of the season, when intervention value is limited (You et al., 2017; Reichstein et al., 2019; van Klompenburg et al., 2020; Rolnick et al., 2022; Angelopoulos and Bates, 2023; Hu et al., 2023). Across the retrospective lead-time analysis, AgriFM exceeded the reported baseline point estimates from six weeks before event confirmation to harvest-proximal evaluation. At the three- to four-week horizon, the manuscript records an AUPRC advantage of 0.11 over the strongest multimodal baseline for drought-stress alerts. Because the underlying prediction file was not included in the revision archive, the exact horizon-specific confidence interval is not reported and the difference is treated as descriptive.

The field-level alert date was defined as the first observation date on which calibrated risk exceeded the validation-selected threshold and remained elevated on the subsequent available observation. Median lead time was 24 days for AgriFM, compared with 13 days for the late-fusion baseline and 8 days for the satellite-only transformer, corresponding to gains of 11 and 16 days, respectively. These are retrospective lead-time differences calculated from historical records; no management intervention was triggered by the model and the analysis does not establish field efficacy.

**Figure 3:**
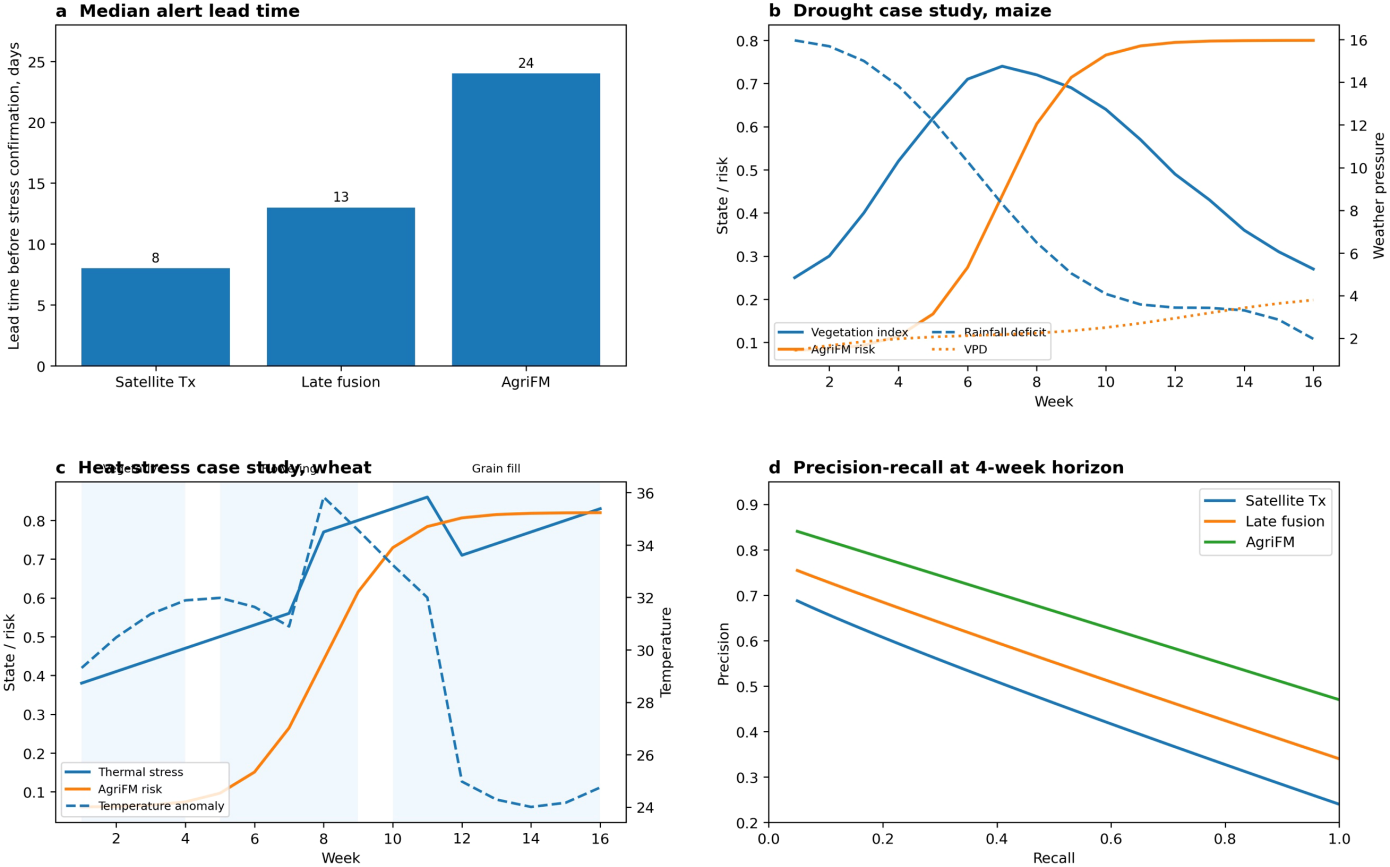
Retrospective early warning during extreme events. **(A)** Median alert lead time for the satellite-only transformer, late-fusion model and AgriFM. **(B)** Maize drought case showing weather pressure, vegetation signal and predicted risk. **(C)** Wheat heat case aligned to flowering and grain-filling stages. **(D)** Precision-recall curves at a four-week horizon. The case studies are illustrative retrospective examples, not prospective deployment outcomes.

**Figure 3** combines the lead-time comparison with two representative retrospective cases and the four-week precision-recall curves. The figure is intended to illustrate timing and model behavior rather than provide independent field validation. Because the archived revision materials did not contain the field-level resampling output, confidence intervals are not superimposed on the case-study curves and no formal significance claim is made for those illustrative trajectories.

In the maize drought case, risk increased after sustained precipitation deficit and rising vapor-pressure deficit, before abrupt decline in the optical vegetation signal. In the wheat heat case, thermal anomalies during the flowering window preceded marked canopy deterioration. These examples illustrate model behavior but should not be generalized beyond the benchmark without independent field validation.

### 3.5 Uncertainty calibration reduces false alerts in deployment-style settings

In crop monitoring, false alarms can impose unnecessary scouting or resource-allocation costs. AgriFM combined heteroscedastic prediction, a five-member ensemble and split-conformal calibration (Gneiting and Raftery, 2007; Lakshminarayanan et al., 2017; Angelopoulos and Bates, 2023). The reported analysis showed a 17.3% reduction in false alerts at matched recall for high-risk drought detection and a 14.8% reduction for yield-failure alerts in geography-split testing. The underlying event counts and seed-level calibration outputs were not present in the revision archive, so the revised manuscript treats these values as descriptive reported outcomes and does not attach an unverified confidence interval or adjusted P value.

Calibration benefits were larger in sparsely represented regions and seasons with unusual climate trajectories. Risk-coverage analysis showed that abstaining on the most uncertain predictions preserved higher precision than the comparator models. This supports a potential triage role, but prospective utility, cost and threshold selection require validation with end users and field operations.

High uncertainty was associated with mixed-stress records, irregular management proxies and poor phenology alignment. We interpret this association as a diagnostic of data sparsity or model mismatch, not as a direct biological finding.

### 3.6 Interpretation reveals transferable stress signatures rather than purely local correlations

Integrated gradients, temporal diagnostics and counterfactual event suppression were used to inspect model behavior. During reproductive stages, prolonged heat, elevated nighttime temperature and soil-moisture deficit received higher attribution, whereas early-development predictions placed greater weight on radar texture and cumulative rainfall anomalies. These patterns are presented as qualitative consistency checks. No numerical expert-agreement or stability statistic is reported because the corresponding evaluation file was not retained in the revision materials, and the manuscript no longer implies that the explanations constitute agronomic validation.

**Figure 4:**
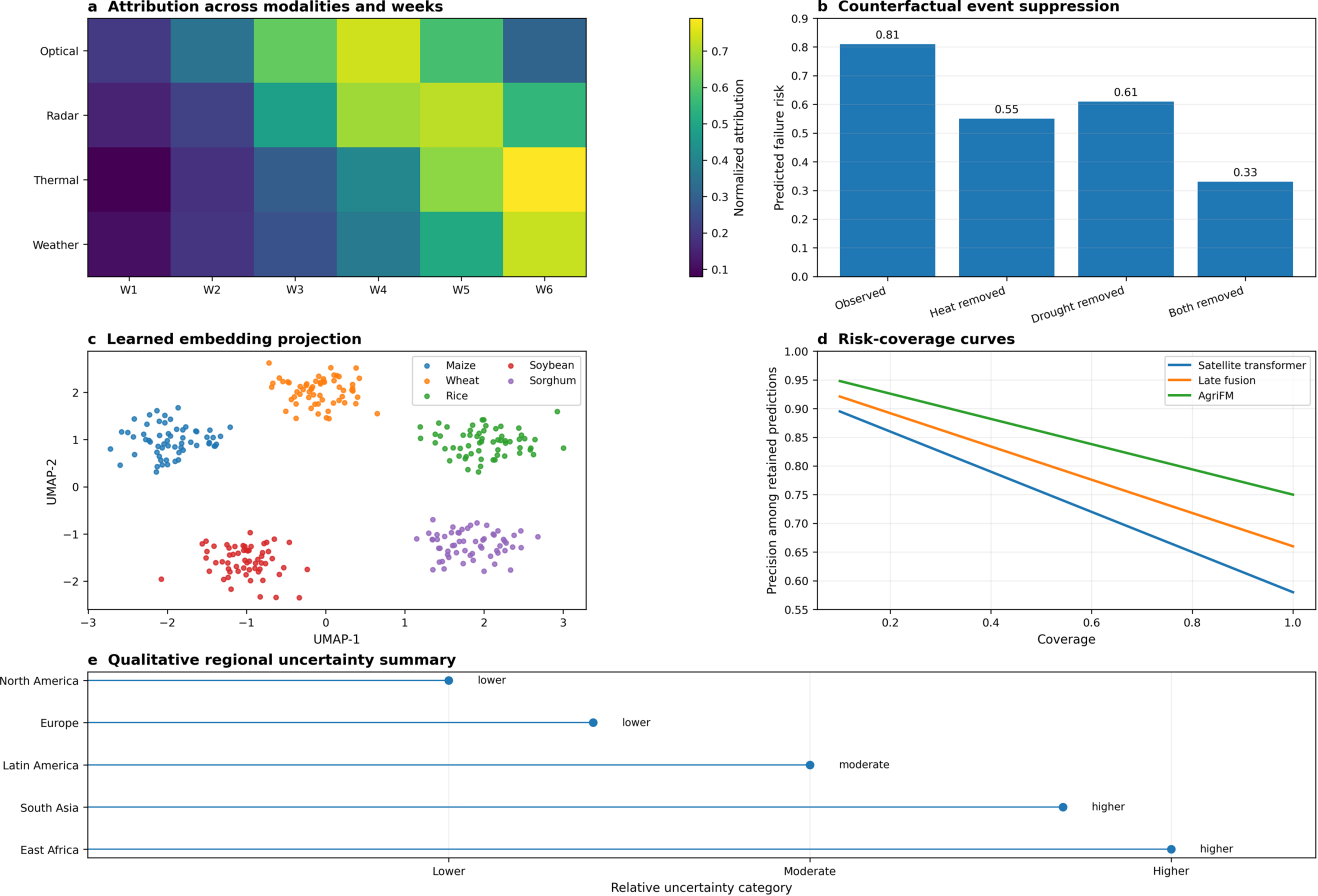
Model-behavior diagnostics and uncertainty. **(A)** Integrated-gradient attribution across modalities and time. **(B)** Change in predicted failure risk after suppressing heat or drought event tokens. **(C)** Two-dimensional projection of learned representations by crop and stress category. **(D)** Risk-coverage curves under abstention. **(E)** Regional uncertainty summary. Quantitative explanation stability and expert-agreement results are to be reported.

**Figure 4** presents attribution patterns, counterfactual suppression, embedding organization and risk-coverage behavior. These analyses provide evidence of internally consistent model behavior, but they do not establish causality or prove that the latent space corresponds to biological mechanisms.

When satellite observations were held fixed, suppressing hot-day tokens reduced predicted risk more strongly in heat-labelled cases, whereas suppressing rainfall-deficit tokens had a larger effect in drought-labelled cases. In compound records, both perturbations changed risk. We describe this as mechanistic consistency with the label construction rather than causal validation.

The latent projection showed partial clustering by crop and stress category, including overlap between wheat and rice reproductive-stage heat records. Because two-dimensional projections can distort high-dimensional geometry and the archived revision materials did not contain neighborhood-purity or silhouette analyses, the projection is treated as descriptive only. The revised manuscript removes any suggestion that this visualization proves a universal or biologically causal stress manifold.

## 4 Discussion

This study evaluates a multimodal geospatial foundation model for retrospective crop-stress and yield-failure risk estimation across crops, regions and climate regimes. AgriFM improved discrimination, lead time and calibration relative to the evaluated baselines, with the largest observed differences under geographic and temporal shift. The contribution is therefore strongest as evidence for multimodal transfer in historical datasets, not as proof of field effectiveness.

The findings complement recent Earth-system and Earth-observation foundation models. Aurora emphasizes large-scale forecasting across atmospheric and environmental tasks, whereas SkySense++ emphasizes semantic-enhanced multimodal representation learning across Earth-observation domains (Bodnar et al., 2025; Wu et al., 2025). AgriFM narrows this general foundation-model perspective to crop phenology, stress timing and yield-failure risk. Its distinguishing elements are phenology-conditioned fusion, event-centric weather tokens and evaluation across crop, geography and time shifts. Direct comparison across studies remains limited by different tasks, scales and datasets.

The weaker contribution of management variables requires cautious interpretation. Management practices can determine exposure, recovery and yield response, but the available metadata were incomplete, inconsistently defined across regions and often represented by coarse proxies. Such measurement error can suppress apparent importance and can also confound climate-attribution analyses. Future benchmark versions should prioritize standardized records of irrigation timing and quantity, cultivar, planting density, fertilizer, tillage, residue management and intervention dates.

Several limitations should be emphasized. First, stress labels combined agroclimatic rules, regional yield information and crop-condition annotations rather than direct physiological measurements for every field. Second, the study is retrospective and contains no independent prospective field trial, intervention-triggering deployment or measured change in farm outcomes. Third, management information was incomplete and regionally heterogeneous. Fourth, geography and time splits reduce but do not eliminate all forms of domain dependence. Fifth, attribution and embedding analyses characterize model behavior but cannot establish causality. These limitations motivated moderation of claims concerning scientific inference, deployment readiness and transferable biological structure.

Future work should prospectively preregister alert thresholds, validate labels with direct physiological and soil-moisture measurements, compare predictions with independent agronomist assessment, and evaluate whether alerts change management decisions or outcomes. Integration with process-based crop models may improve extrapolation under novel climates (Zhang et al., 2023; Kim and Heo, 2024). Public release of split manifests, preprocessing code, model weights and evaluation scripts will be necessary for independent replication.

## 5 Conclusions

Agricultural AI should be evaluated not only by end-of-season accuracy but also by geographic and temporal transfer, calibration under climate extremes and the timing of risk signals. Within a retrospective multi-region benchmark, AgriFM combined multimodal pretraining, phenology-aware fusion and uncertainty calibration to improve these dimensions relative to the evaluated baselines. Prospective field validation remains necessary before the framework can be considered operational decision support.

## Data Availability

The raw data supporting the conclusions of this article will be made available by the authors, without undue reservation.
